# Draft Genome Sequence of the Biofilm-Forming *Stenotrophomonas maltophilia* Strain 53

**DOI:** 10.1128/genomeA.00312-15

**Published:** 2015-04-16

**Authors:** Sirwan Akbar, Simon P. Rout, Paul N. Humphreys

**Affiliations:** Department of Biological Sciences, School of Applied Sciences, University of Huddersfield, Huddersfield, United Kingdom

## Abstract

A clinical strain of *Stenotrophomonas maltophilia* (designated strain 53) was obtained, and a whole-genome sequence was generated. The subsequent draft whole-genome sequence demonstrated the presence of a number of genes encoding for proteins involved in resistance to a number of antimicrobial therapies.

## GENOME ANNOUNCEMENT

*Stenotrophomonas maltophilia* is an aerobic, Gram-negative, and nonfermentative microorganism, originally described as *Bacterium booker* in 1943 before being placed in the novel genus *Stenotrophomonas* in 1993 (1). Strains of *Stenotrophomonas maltophilia* have been found almost ubiquitously within natural environments, including freshwater sources, and within both the soil and plant microbiomes (2–4). This species has received attention with regard to its bioremediation properties, where the degradation of a number of contaminants has been described (5, 6). Within a clinical setting, members of this species also represent a risk of nosocomial infection, where it is an opportunistic pathogen to immunosuppressed or debilitated individuals (7, 8). Mechanisms such as decreased permeability, β-lactamases, aminoglycoside-modifying enzymes, and the presence of efflux pumps within the outer membrane aid this pathogenicity (9). This resistance appears to be mediated not only by intrinsic resistance mechanisms but also through its ability to acquire resistance via uptake and integration of genetic information into the host chromosome (10).

A hospital isolate of *Stenotrophomonas maltophilia* was obtained and subcultured into tryptone soya broth. Following incubation for 24 h at 37°C, total genomic DNA was isolated using a commercial kit (Ultraclean microbial isolation kit; Mo-Bio, USA). A draft whole-genome sequence was obtained using a whole-genome shotgun sequence strategy. Paired-end 125-cycle sequence reads were generated using Illumina HiSeq 2500 technology (BaseClear, Netherlands). FASTQ sequence files were generated using the Illumina CASAVA pipeline version 1.8.3, and the CLC Genomics Workbench version 7.0 was used for assembly. The contigs were linked and placed into scaffolds or supercontigs. The orientation, order, and distance between the contigs were estimated using the insert size between the paired-end and/or mate-pair reads using the SSPACE Premium scaffolder version 2.3 (11). Whole-genome sequencing generated 128 contigs with a draft genome 4,637,878 bp in length and a G+C content of 66.3%. The draft genome contained a total of 4,082 coding sequences (CDS), where 77 pseudogenes, 3 genes coding for rRNA (5S, 16S, 23S), 64 genes coding for tRNAs, and 1 noncoding RNA were present. RAST (12) annotation showed that 31 genes were present encoding multidrug-resistance efflux pumps, 21 were involved in cobalt-zinc-cadmium resistance and 10 genes were involved in the production of β-lactamases. Also present were genes involved in resistance to arsenic and fluoroquinolones, as well as tripartite efflux pumps and a single gene encoding an aminoglycoside adenylyltransferase. In addition, genes were present that were responsible for encoding proteins involved in the synthesis of biofilms, which are likely to enhance its pathogenicity (13). Overall, the strain demonstrates the potential for resistance to a wide range of antimicrobial strategies within the clinical setting.

### Nucleotide sequence accession number. 

This whole-genome shotgun project has been deposited at DDBJ/EMBL/GenBank under the accession number JRJA00000000.
